# The Brain as an Organ of Mind

**Published:** 1880-10

**Authors:** 


The Brain as an Organ of Mind.—By H. Charlton Bastain, M.A., M.D., F. R. S., Professor of Pathological Anatomy, and of Clinical Medicine in University College of London, Etc. With 184 illustrations. D. Appleton & Co., New York. 1880.

Conspectus of Organic Materia Medica and Pharmacal Botany, comprising the vegetable and animal drugs, their physical character, geographical origin, classification, doses, adulteration, etc., etc.

